# Towards Facial Gesture Recognition in Photographs of Patients with Facial Palsy

**DOI:** 10.3390/healthcare10040659

**Published:** 2022-03-31

**Authors:** Gemma S. Parra-Dominguez, Raul E. Sanchez-Yanez, Carlos H. Garcia-Capulin

**Affiliations:** Department of Electronics Engineering, Universidad de Guanajuato DICIS, Salamanca 36885, Mexico; gs.parradominguez@ugto.mx (G.S.P.-D.); carlosg@ugto.mx (C.H.G.-C.)

**Keywords:** facial gesture recognition, facial expression recognition, FER systems, facial palsy, facial paralysis

## Abstract

Humans express their emotions verbally and through actions, and hence emotions play a fundamental role in facial expressions and body gestures. Facial expression recognition is a popular topic in security, healthcare, entertainment, advertisement, education, and robotics. Detecting facial expressions via gesture recognition is a complex and challenging problem, especially in persons who suffer face impairments, such as patients with facial paralysis. Facial palsy or paralysis refers to the incapacity to move the facial muscles on one or both sides of the face. This work proposes a methodology based on neural networks and handcrafted features to recognize six gestures in patients with facial palsy. The proposed facial palsy gesture recognition system is designed and evaluated on a publicly available database with good results as a first attempt to perform this task in the medical field. We conclude that, to recognize facial gestures in patients with facial paralysis, the severity of the damage has to be considered because paralyzed organs exhibit different behavior than do healthy ones, and any recognition system must be capable of discerning these behaviors.

## 1. Introduction

The human ability to effectively communicate emotion is essential to perform daily activities, and it is required for personal and social sufficiency. Humans express their emotions not only verbally but also through their actions. It has been shown that 93% of communication is non-verbal; thus, facial expressions and body gestures play an essential role in it [1]. This is why emotion recognition systems use facial expressions, speech, body gestures, physiological signals, bio-signals body movements, and other information to identify emotional states [2]. Depending on the sensors and features extracted to detect emotion, recognition systems are unimodal or multimodal. Some systems specifically operate using only information extracted from facial expressions [2], and those are of particular interest for this research.

Of all the ways that humans communicate emotion, facial expressions are among the most flexible, and their universality allows us to rapidly convey information to people of different ages, cultures, and languages [3]. This is the reason why gesture and facial expression recognition has recently become a popular topic with applications in various fields, for example, in security, healthcare, entertainment, advertisement, education, and robotics [4]. Computer vision has reached high accuracy in the automatic recognition of facial expressions; extensive research is found in [2,4,5,6].

**Figure 1:**
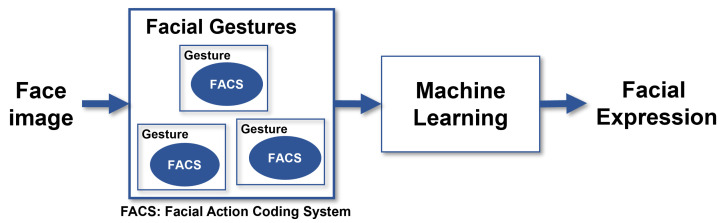
A common facial expression recognition system based on the analysis of face images and machine-learning algorithms to output a facial expression.

A facial expression can be represented by one or more gestures, as shown in Figure 1; identifying those could help discriminate one expression from another. Facial Expression Recognition (FER) systems are computer-based technology that uses mathematical algorithms to analyze faces in images or video [7,8].

The analysis of facial expressions allows us to classify them into emotion categories; in other words, giving meaning to a facial expression permits us to recognize and label it as anger, disgust, fear, happiness, sadness, surprise, contempt, etc. For the sake of clarification, it is essential to mention that, in the literature, the acronym FER often refers to facial expression recognition or facial emotion recognition [7].

The difference between both depends on the system’s primary objective; usually, an algorithm designed to recognize emotions first, performs facial expression recognition tasks, among others, and it is said that the recognition of emotional states is based on facial expressions. FER stands for facial expression recognition in this work because this study’s principal interest is to classify face gestures before achieving facial expression recognition.

Detecting facial expressions continues to be a complex and challenging problem [5]. In general, a camera-based FER system operates in three primary stages: preprocessing, feature extraction, and classification [9]. In the preprocessing stage, the main step is face detection within the image. Face detection is complex because it is affected by face gestures, poses, and lighting conditions. Feature extraction depends on the type of FER system, whether frame-based or sequence-based. The first refers to a system that considers a single frame to distinguish among facial expressions, and the second extracts temporal information to detect expressions from a set of frames [10].

FER systems can also be classified as appearance or geometric-based methods. Appearance-based methods extract features from textural information of the face. In contrast, geometric-based methods rely on features computed from the shape change information of the face during expressions [11]. A number of machine-learning algorithms are used to classify the subject’s expression and emotion accurately. Some popular classifiers are the linear discriminant classifiers, k-nearest neighbor, Gaussian mixture model, support vector machines, artificial neural networks, deep neural networks, decision tree algorithms, and hidden Markov models [2].

Emotion detection is currently a popular research area in computer science fields. However, the face analysis for medical and health applications is still in an embryonic state [3]. Applications in the medical field are designed to reduce the patient’s stress, depression, and anxiety. In other words, automated systems are being developed to recognize their emotions and then provide appropriate therapy to manage any adverse reaction in the patient [2].

According to Leo et al. [3], the healthcare frameworks, which include an emotion or expression recognition module, have been introduced to provide suitable solutions for the following: (1) long-term care for person-centered and integrated healthcare systems; (2) diagnosis or assessment of cognitive impairments (e.g., autism, schizophrenia, and profound intellectual and multiple disabilities [12]) and automated pain detection; (3) the use of technological rehabilitation frameworks [13,14], and (4) the design of smart environments that react in a friendly manner according to the patient’s necessities.

Yolcu et al. in [11] argued that some neurological disorders show well-known impaired facial expressions. Therefore, it is necessary to design automated systems to efficiently detect emotions in, for example, Parkinson’s, stroke, and facial palsy. In the literature, and to the best of our knowledge, only one recent study aimed to evaluate the emotions from facial expression in patients with facial palsy. Xu et al. in [15] stated that the automatic recognition of emotions could be a solution to help understand facial palsy patients, acknowledge their stress in advance, and assist their treatment. The authors also stated that their facial expressions are different from healthy subjects due to the inability of the facial muscles, making the existing data and models from healthy people invalid [15].

Facial paralysis or facial palsy describes the incapacity of the face to move its muscles on one or both sides, as previously mentioned. This incapacity can originate from nerve damage due to congenital conditions, trauma, or diseases (e.g., stroke, Bell’s palsy or brain tumor). There is a noticeable drooping of the facial features and problems with ordinary activities, such as speaking, blinking, swallowing saliva, eating, and communicating through natural facial expressions. In general, the diagnosis of facial palsy is not difficult; it only requires a healthcare professional to visually inspect the patient’s facial symmetry and determine the presence and level of paralysis. Since this inspection requires specific medical training to produce a diagnosis, automatic approaches to diagnosing and evaluating facial palsy have been developed in recent years.

The wide variety of methodologies working with facial palsy found in the literature can be divided according to the primary task they perform. For example, the detection of paralysis via a binary classification between healthy or unhealthy subjects or assessing the patient’s level of paralysis (damage severity). In our previous work [16], a frame-based methodology using geometrical features and neural networks was introduced for detecting facial paralysis on a publicly available database.

This research seeks to classify facial gestures in palsy patients as a first step in recognizing emotions from facial expressions. Other authors have identified the necessity of having specific analysis, data, and models for the detection of facial gestures and expressions in palsy patients [11,15,17], different from the actual work developed with and performed on healthy people. The main contributions of this work are (1) an analysis of facial gestures in persons with facial palsy, (2) a system for the recognition of gestures on photographs of patients exhibiting facial paralysis (such a system consists of a neural network using handcrafted features extracted from facial landmarks), and (3) evaluations on a publicly available database.

The remainder of the paper is organized as follows: Section 2 describes the proposed methodology. Section 3 describes the database employed in this research and introduces the experiments, findings, and discussion. Finally, Section 4 provides the concluding remarks.

## 2. Methodology

This research assumes that a facial expression is composed of one or more facial gestures, and a gesture is produced by activating one or more muscles. In computer vision, it is widely accepted that muscle activity is defined by action units (AUs); in the field of face analysis, actions units are encoded in the Facial Action Coding System (FACS) described by Kanade et al. in [18] and later in [19]. Identifying specific FACS led us to recognize specific facial gestures as depicted in Figure 1 and, later, to classify facial expressions [4].

**Figure 2:**
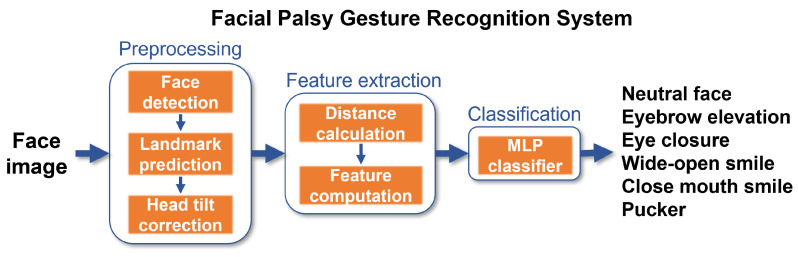
Framework of the proposed facial palsy gesture recognition system.

The proposed methodology is frame-based, meaning that it inspects a single image aiming to recognize the facial gesture within it. This approach performs face detection and then predicts facial landmarks. The geometrical features are computed from those landmarks mainly in three specific regions: the eyebrows, eyes, and the mouth, similarly to other approaches [3]. A multi-layer perceptron is then trained to detect six gestures: rest (neutral), eyebrow elevation, eye closure, wide-open smile, closed mouth smile, and puckered face, as shown in Figure 2.

A description of the analyzed facial gestures is introduced in Table 1, divided into three facial regions and the more relevant AUs involved on each. In some cases, not all the AUs are meaningful to recognize the gesture; then, they are marked as irrelevant for the methodology. It does not mean they are not relevant in a different context. Further description of the method is provided next.

### 2.1. Step 1: Preprocessing

**Figure 3:**
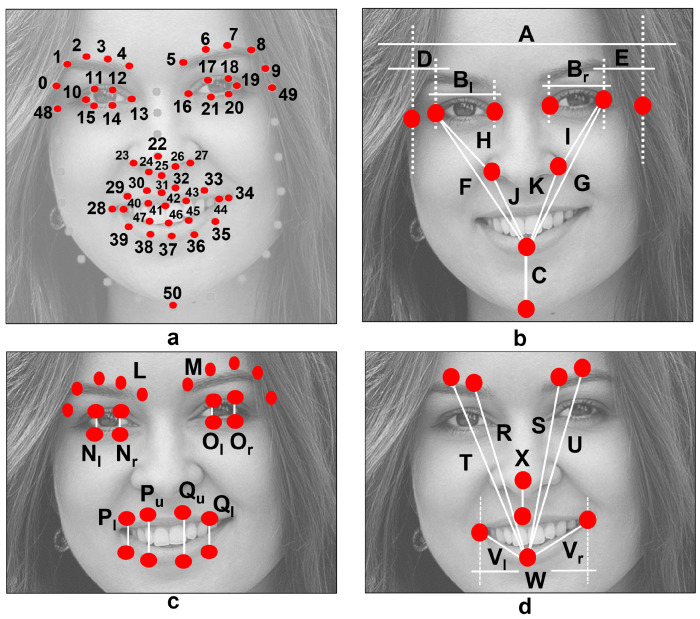
(**a**) The 51 key points inspired in the 68-point model proposed by Matthews and Baker [23]. (**b**–**d**) Facial distances to compute spatial relations between facial landmarks [16].

The first step in our gesture recognition system includes face detection and the prediction of facial landmarks. Color conversion to grayscale and image resize is performed prior to detect the face using the dlib C++ Library [20]. Facial landmarks are predicted using the shape predictor proposed and introduced in [21]. Note that the predictor has been trained to detect 68 key points; however, only 51 are of interest. These were reorganized and are shown in Figure 3a. The full process is described in [16].

A tilt correction of the head’s angle is performed using the landmarks previously predicted. It has been demonstrated in [16,22] that such a tilt angle can affect the computation of measures. To suppress this influence, a rotation of the predicted landmarks is performed using a transformation matrix. This transformation matrix is calculated according to the similarity transform approach and four known point coordinates. The process goes as follows:

1. Set the outer eyes’ corners as point coordinates. These are landmark 10 (left corner of the left eye) and landmark 19 (right corner of the right eye) in Figure 3a.
2. Determine a new set of point coordinates, which are horizontally aligned and represent the new position of the outer eyes’ corners.
3. Calculate the transformation matrix $T_{f}$ according to the similarity transform approach.
4. Transform the face image using $T_{f}$ and the affine transformation approach (optional).
5. Rotate the predicted landmarks using $T_{f}$.

The landmark rotation process can be performed with a multiplication of matrices, as stated in Equation (1)

(1) $$\left(\begin{array}{c} Pir_{x}\\ Pir_{y} \end{array}\right)=\left[\begin{array}{ccc} T_{f(1,1)} & T_{f(1,2)} & T_{f(1,3)}\\ T_{f(2,1)} & T_{f(2,2)} & T_{f(2,3)} \end{array}\right]\left[\begin{array}{c} Pi_{x}\\ Pi_{y}\\ 1 \end{array}\right]$$

### 2.2. Step 2: Feature Extraction

When working with facial palsy patients, the asymmetry of the face must be considered. Usually, the main difference is observed between the left and right sides of the face, but others can be observed depending on the disease or the affected nerves. The proposed methodology mainly compares and quantifies the differences between the left and right sides by analyzing the location and position of the face organs (eyebrows, eyes, nose, and mouth). Initial tests concluded that the regions of the eyebrows, eyes, and mouth provide the most meaningful information for this challenge, and this is similar to the findings of other authors [22,24,25,26].

In this research, a total of 30 measures (28 distances and two average values) are calculated using the extracted key points, as depicted in Figure 3b–d. Distances A to K are inspired by the work of Ostrofsky et al. in [27], where the authors evaluated objective measures from face photographs with a target other than the analysis of facial palsy. Still, they appear to be an excellent reference to characterize the human face. The rest of the distances (L to W) in Figure 3c,d, were found to be helpful in the previous work [16], which aimed to detect facial paralysis in photographs.

Features were computed using the aforementioned 30 measures and, after several evaluations, 29 were found helpful in detecting a level of asymmetry within the face and performing gesture recognition tasks. The 29 features are described in Table 2. Please refer to Figure 3 and to [16] if further detail is required. These features were extracted as follows: using Equation (2) to obtain the angle between two points, Equation (3) to calculate the slope between points, Equation (4) to compute the Euclidean distance, and Equation (5) to calculate the perimeter of a closed shape.

(2) ∠(Pa,Pb)=arctan2(▵x,▵y)×180/π

where $▵x=Pa_{x}-Pb_{x}$ and $▵y=Pa_{y}-Pb_{y}$.

(3) $$m\left(Pa,Pb\right)=\left|\frac{Pa_{y}-Pb_{y}}{Pa_{x}-Pb_{x}}\right|$$

(4) $$d\left(Pa,Pb\right)=\sqrt{{\left(Pa_{x}-Pb_{x}\right)}^{2}+{\left(Pa_{y}-Pb_{y}\right)}^{2}}$$

(5) $$\bar{S}\left(P_{s},\ldots ,P_{l}\right)=\sum_{x=s}^{l-1}d\left(P_{x},P_{x}+1\right)+d\left(P_{s},P_{l}\right)$$

where $\bar{S}$ is a closed shape, $P_{s}$ is the start point, and $P_{l}$ is the last one and represents the end point within the shape.

**Figure 4:**
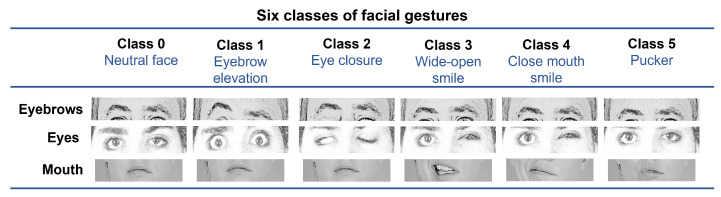
The proposed facial gestures grouped into six classes.

In this research, the proposed features are also capable of discriminating between different positions and shapes of the face organs involved in the gestures described in Table 1. Figure 4 intends to show the behaviors that these elements reveal for each gesture. For the eyebrows, the most significant asymmetry is observed with the eyebrow elevation movement. Depending on the palsy severity, such asymmetry can also be observed in other expressions, for example, while resting the face. For the eyes, the most asymmetrical level is found for the eye closure; however, a significant change is also observed when the subject elevates the eyebrows.

We observed that when the patient is asked to raise the eyebrows, the movement comes with a whole opening of the eyes; the mouth is irrelevant in this case. Smiling shows the most considerable asymmetry within the lips, vertically in the case of a wide-open smile and horizontally in the other case. Here, it is also observed that patients tend to show a slight asymmetry in the eyes opening when smiling and when performing a pucker gesture.

Depending on the palsy severity, this asymmetrical eye-opening could be observed when resting the face. Notice that the photographs represent posed expressions; a different behavior could be observed in in-the-wild images. In Figure 4, the reader can infer that the proposed methodology aims to discriminate among six classes (i.e., rest, eyebrow elevation, eye closure, wide-open smile, closed mouth smile, and pucker).

### 2.3. Step 3: Classification

The Waikato Environment for Knowledge Analysis (Weka) was employed to achieve the proposed facial gesture classification task. Weka is a platform for general-purpose machine-learning tasks, such as classification, prediction, detection, etc. Weka is a graphical user interface that contains the full pipeline for managing data sets, training, and testing according to multiple machine-learning models; therefore, it was appropriate for the analysis performed in this research. For more information regarding the Weka platform, please refer to [28].

Particularly, the Weka function known as the Multilayer Perceptron (MLP) was used to train and test a multi-class classifier based on the multi-layer perceptron strategy. This MLP classifier requires a few parameters to learn. Some of those can be easily set up in the Weka environment, for example, the learning rate (L), momentum (M), training time (N), number of neurons in each of the layers (input, hidden, and output) (H), and seed (S). During the training phase of the MLP strategy, the learning rate and the momentum are required to update the weights of the connections between network nodes. As indicated by its name, the training time refers to the number of iterations to train through, and the value in H refers to the number of neurons in each layer within the network. Finally, the value of S refers to a number employed to initialize the network’s weights randomly. The MLP strategy uses backpropagation to learn and classify instances. More information regarding the Weka software may be found at [28].

It is relevant to mention that the Weka suite operates using Attribute-Relation File Format (ARFF) files, which are text files that describe a list of instances (samples) sharing a set of attributes (features and labels); information on how to create ARFF files can be found in [29]. ARFF files are created for the training and testing sets in this work, those files are later loaded into Weka, and the training and evaluation process begins. Next, further details on the experiment and results are provided.

## 3. Results and Discussion

Several methodologies are proposed to detect facial paralysis in a photograph; however, no methods seek to analyze and recognize facial gestures in patients with palsy. To our knowledge, annotated data indicating the patient’s emotional state is not publicly available. This is likely because the available data is limited due to patients’ privacy; under this scenario, we decided to design a recognition system using a publicly available dataset called the Massachusetts Eye and Ear Infirmary (MEEI) database. This image database was collected by Greene et al. and introduced in [17], the MEEI is an open-source set of face photographs and videos representing the whole flaccid and nonflaccid facial palsy spectrum. The database was launched with the purpose to serve as a resource for facial paralysis research and education; some works that have employed the MEEI database are [16,21,30,31].

The relationship between the level of facial function and the perceived emotion in a palsy patient was characterized by Greene et al. in [17] using a machine-learning algorithm to initially demonstrate the utility of the MEEI database. That particular algorithm was designed and trained using healthy people imagery, and then, the recognition of the communicated emotion from the palsy patients was inaccurate as concluded by the authors themselves in [17].

The MEEI dataset consists of 480 high-resolution images from 60 subjects: ten healthy participants, 25 patients with flaccid palsy, and 25 patients with nonflaccid paralysis. Each subject performed eight well-known facial movements: (1) at rest, (2) eyebrow elevation, (3) light effort eye closure, (4) full effort closure, (5) light effort smile, (6) full effort smile, (7) pucker, and (8) lip depression. The MEEI database is also annotated according to the level of paralysis; in other words, six palsy grades are provided: normal, near-normal, mild, moderate, severe, and complete. It is relevant to mention that those facial grading scores seek to evaluate the level of paralysis rather than perform emotion recognition tasks. The scope of our research only aims to recognize facial expressions with this image database.

The eight facial movements are grouped into six classes in the proposed approach, as previously shown in Figure 4. Closing the eyes is considered a single gesture since it is not the intention to measure the intensity of the movement, which would be out of the scope of this work. Smiling is split into smiles showing the teeth (wide-open smile) or just enlarging the lips (closed mouth smile); again, measuring the intensity of the gesture is out of the scope. During preliminary tests, we observed that, for a palsy patient, performing a lip depression—which consists of showing the lower teeth—is very similar to a wide-open smile; thus, it was grouped as a single facial gesture.

The five-fold cross-validation protocol is adopted to test the model accuracy. This allows testing on unseen images, thus, reducing the possibility of over-fitting to previously seen ones. The five-fold cross-validation method divides the data set into five subsets. Every single subset is retained as validation data. The other four are used as training data, ensuring the test data is untouched in each experiment session. The experiment is repeated five times, and each subset has the same probability for validation. The accuracy, recall, precision, and F1 score values are computed as evaluation metrics according to (6), (8), (7), and (9), respectively.

(6) $$\mathrm{Accuracy}=\frac{\mathrm{TP}+\mathrm{TN}}{\mathrm{TP}+\mathrm{TN}+\mathrm{FP}+\mathrm{FN}}$$

(7) $$\mathrm{Recall}=\frac{\mathrm{TP}}{\mathrm{TP}+\mathrm{FN}}$$

(8) $$\mathrm{Precision}=\frac{\mathrm{TP}}{\mathrm{TP}+\mathrm{FP}}$$

(9) $$F1-\mathrm{score}=\frac{2\times \mathrm{TP}}{2\times \mathrm{TP}+\mathrm{FP}+\mathrm{FN}}$$

where TP stands for true positives, TN for true negatives, FP for false positives, and FN for false negatives.

Due to the limited amount of data, 80 healthy and 400 palsy samples (200 flaccid and 200 non-flaccid), it was necessary to augment the data set. Following the strategy suggested in [22,32], the samples were rotated in opposite directions, increasing the amount of data and also verifying that the proposed methodology is invariant to rotation. In preliminary tests, increasing the amount of data to 560 healthy and 2800 palsy samples, meaning three rotations in opposite directions and the original data, was shown to be sufficient to obtain good results without over-fitting.

### 3.1. Performance Evaluation

As previously stated, the classification task (i.e., facial gesture recognition) was performed using the MLP approach with the *L* and *M* parameters set as previously proposed in our work (i.e., L=0.2045 and M=0.1909) [16]. The rest of the parameters were defined after several trials and a value optimization process using the Weka platform. We found that H=95, S=0, and N=5000 yielded the best results for the system. The entire data set was used during the design of the classifier; 80% of the samples were used to train and 20% to test according to the five-fold cross-validation strategy, as mentioned earlier.

It is out of the scope of this research but to provide a reference of the necessity of having new models designed with palsy data, a classifier was trained and tested using only the healthy data from the MEEI database. The 560 samples were distributed as follows: 70 samples for class 0, 70 samples for class 1, 140 samples for class 2, 161 samples for class 3, 49 samples for class 4, and 70 samples for class 5. The overall accuracy of 90.71% was reached in recognizing facial gestures using the five-fold cross-validation strategy. The palsy data was also evaluated with this classifier trained with healthy samples, and the performance decreased to 58.96%. This shows that a model trained with palsy information is required, as discussed earlier.

Another classifier was trained using the 2800 palsy samples, which are distributed as follows: 350 samples for class 0, 350 samples for class 1, 700 samples for class 2, 868 samples for class 3, 182 samples for class 4, and 350 samples for class 5. The five-fold cross-validation results are provided in Table 3, where the values correspond to the performance of the testing part of the data for each fold. The system accuracy is 90.25% with the best result of 90.86% and the worst of 89.46%.

The results provided in Table 4 reflect the capacity of the system to recognize each class. It is easy to observe that the best-recognized gesture is class 3 (wide-open smile), while class 4 (close-mouth smile) is the worst in terms of recall values, likely because of the data distribution previously described. A deeper analysis of the capacity of the system to recognize each class is provided in Section 3.2.

**Figure 5:**
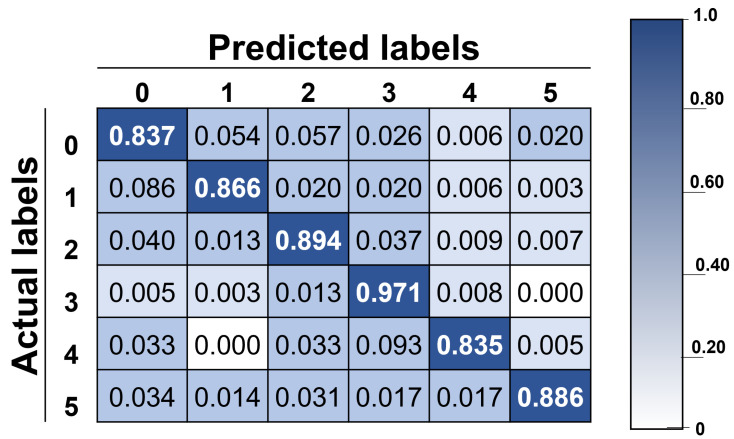
Confusion matrix with actual and predicted labels: class 0 for neutral face, class 1 for eyebrow elevation, class 2 for eye closure, class 3 for wide-open smile, class 4 for closed mouth smile, and class 5 for pucker.

The confusion matrix with actual and predicted labels is introduced in Figure 5, showing the accuracy of the proposed methodology, and by looking into it, it can be observed that the classification system can recognize different facial gestures. The best result is 97.12% for class 3, and the worst is 83.52% for class 4. As mentioned before, the system’s overall accuracy is 90.25%, representing a good result if considering this is a six-class classification system. Furthermore, considering that the baseline accuracy was set at 58.96%, a significant improvement was achieved.

Additional experiments were performed to observe the capacity of the recognition system based on the level of facial palsy. For this test, six classifiers were trained under the same parameters (i.e., L=0.2045 and M=0.1909, H=95, S=0, and N=5000); however, only the data corresponding to the same grade of paralysis was employed. The data distribution consists of 10 participants for each grade: normal, near-normal, mild, moderate, severe, and complete.

The results for the recognition system based on the level of facial palsy are shown in Table 5. Good results are revealed, especially for the complete level of paralysis with an overall accuracy of 96.79%. The lowest performance was 87.14% for the mild grade; this palsy level was the hardest to characterize for our system. The overall accuracy of 90.71% seems acceptable for the normal subjects; however, in the literature highest values of accuracy are found. However, it is not the scope of this research to perform facial expression recognition on healthy people.

**Figure 6:**
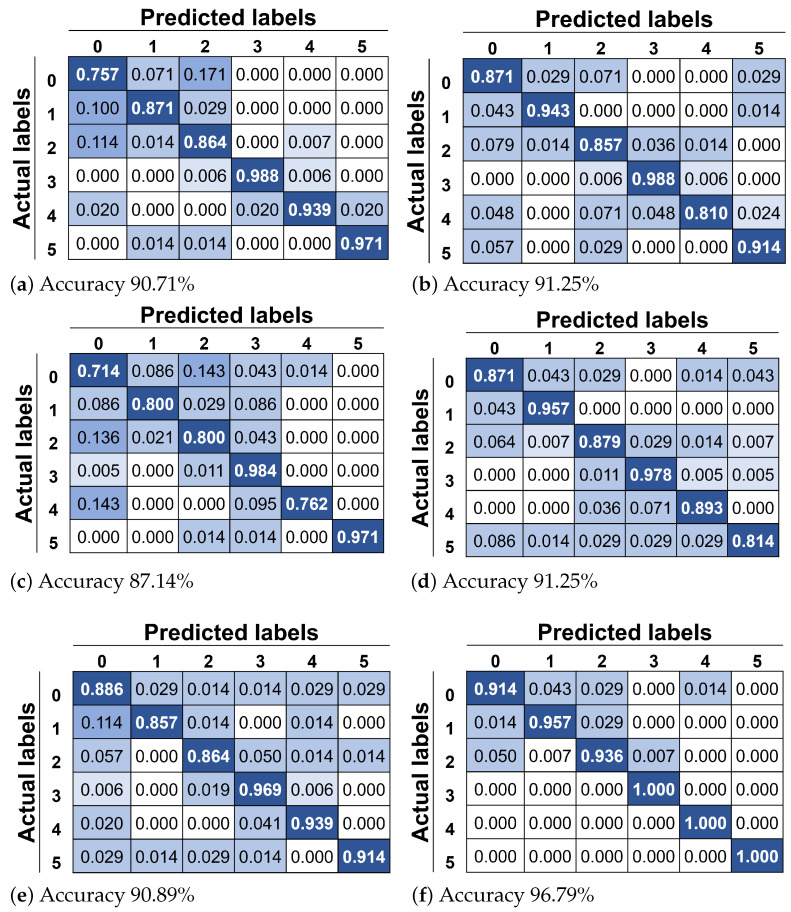
Confusion matrix with actual and predicted labels for test: (**a**) normal, (**b**) near-normal, (**c**) mild, (**d**) moderate, (**e**) severe, and (**f**) complete paralysis. Class 0 refers to neutral face, class 1 to eyebrow elevation, class 2 to eye closure, class 3 to wide-open smile, class 4 to closed mouth smile, and class 5 to pucker.

In Figure 6, the confusion matrix for each of the evaluations is depicted. In Figure 6a, it is possible to observe that a neutral face is mainly confused with closed eyes in a healthy face; this is an expected behavior since a little change in the organs’ shape would be observed because of the symmetry of the face. The same justification applies for eyebrow elevation being confused with a neutral face. In the case of near-normal palsy shown in Figure 6b, the worst class is closed mouth smile, which is confused with eye closure. We argue that this is produced by the activation of the lip corner puller, making what looks like a smile. In the case of mild palsy shown in Figure 6c, it is easy to observe that this level of paralysis is the most difficult to characterize.

Here, the neutral face is confused with four of the five possible classes. In the case of moderate palsy shown in Figure 6d, the worst class is puckered face, which is confused with all possible classes, likely because the asymmetry of the mouth is not sufficient to characterize it into specific gestures. In the case of severe palsy shown in Figure 6e, the worst class is eyebrow elevation, and we argue that this is produced by the incapacity of the patient to raise the eyebrows. Finally, in the case of complete palsy shown in Figure 6e, the worst class is the neutral face, which is slightly confused with three of the five possible classes.

At the beginning of this paper, we mentioned the work of Xu et al., which aimed to evaluate emotions in patients with facial palsy through deep learning [15]. To start, the authors collected images representing facial expressions of six basic emotions from 45 patients with three levels of facial palsy. The authors reported an increase in the performance from 23.89% to 66.58% after training using the palsy data. Moreover, their emotion recognition accuracy rates were 72.22% for mild, 65.83% for moderate, and 61.67% for severe levels of paralysis. Their work is not a direct comparison to our research; however, they are related because both seek to provide solutions in the analysis from images of facial paralysis. Both works concluded that new models trained with palsy data are required to do so.

### 3.2. Analysis

**Figure 7:**
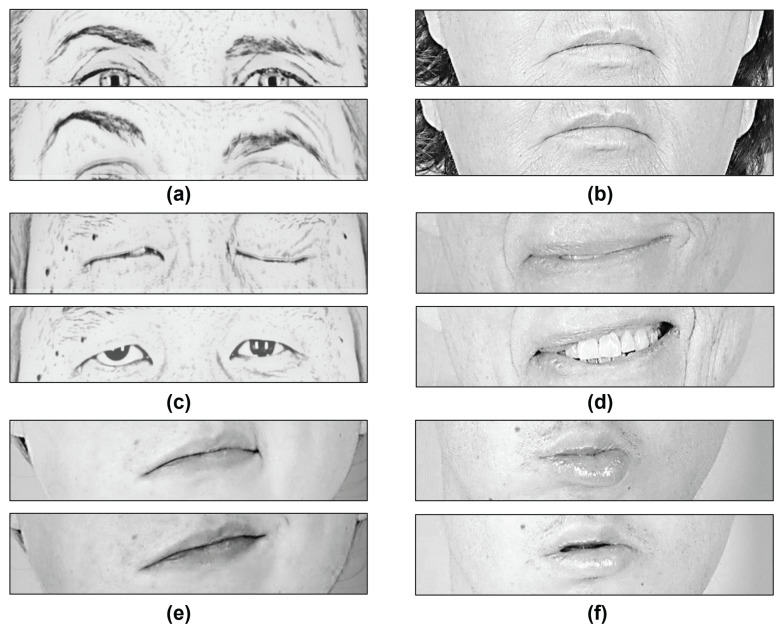
Analysis of facial gestures in patients suffering from facial palsy: (**a**) Small change in the eyebrows’ height. (**b**) Similar mouth shape (width and height). (**c**) Slightly different change in the eyes’ height. (**d**) Similar change in the mouth shape. (**e**) Activated lip corner puller. (**f**) Similar change in the mouth shape.

In this methodology, the levels of asymmetry were found by measuring the change rate of the height and width of each eye and the mouth, and the difference in the vertical position between the eyes and eyebrows. The method mainly compares the left and right sides of the face as depicted in Figure 7, thereby, allowing good results in detecting facial gestures.

In a further analysis and referring to Figure 6 and Figure 7, we found that:

- Neutral face is confused with closing the eyes, because there is a slight difference between the eyebrows’ position (see Figure 7a) and the mouth shape for both gestures (see Figure 7b); however, if there is enough paralysis where one eye remains opened and the other closed, the change is perceived by the classifier and the movement is classified correctly (see Figure 7c).
- Neutral face is confused with the eyebrow elevation; some patients show similar asymmetry in the eyebrows’ vertical position due to their level of paralysis.
- Closed mouth smile is confused with a wide-open smile mainly because a similar rate of change is produced in the eyes opening and in the mouth width or opening (see Figure 7d).
- Closed mouth smile is confused with closing the eyes because, in some patients, a full effort eye closure activates the lip corner puller, producing what looks like a smile (see Figure 7e).
- Closed mouth smile is confused with a neutral face because, in some patients, the asymmetry in the mouth shape is not sufficient to classify it as a smile.
- Pucker is confused with a neutral face because some patients cannot fully perform the gesture. Their mouth looks asymmetrical; however, the eyes and the eyebrows remain close to no movement (see Figure 7f).
- Pucker is confused with closing the eyes because some patients cannot fully perform the gesture but show a change in the eyes’ opening.

The images from the MEEI database are available upon request at https://www.sircharlesbell.com/ (accessed on 1 September 2020).

To achieve facial gesture recognition and later emotion classification in patients with facial paralysis, the level of damage must be considered in the recognition system design because the paralyzed facial organs exhibit different behavior compared with healthy organs. Regarding accuracy and face generalization, improvements to the methodology would require more data from facial palsy patients with labeled facial expressions and emotions.

It is out of the scope of this research; however, it is worth mentioning that a complete facial paralysis system should start with detecting facial palsy in a face photograph (or video, depending on the methodology). Then, a classifier trained on healthy people or palsy patients could be selected for the following tasks depending on the detection result. The next module should include the analysis of facial gestures to recognize facial expressions to detect emotion or any other application involving the face. To date, we proposed a system to detect facial palsy in photographs and now an approach to classify facial gestures. Annotated data, including the patient’s emotional state, is needed for the next step.

## 4. Conclusions

We proposed a methodology based on neural networks and handcrafted features to recognize six gestures in patients with facial paralysis. The proposed gesture recognition system was designed and evaluated on publicly available images from patients with facial palsy. The recognition system obtained an overall accuracy of 90.25%; the best result yielded 97.12% and the worst 83.52%. The achieved performance also shows that our methodology is helpful in solving issues of interest in facial palsy. Other approaches have aimed to analyze faces in subjects with cognitive impairments and pain; however, this research is a first attempt to perform facial gesture recognition in patients with facial palsy.

We found that any facial gesture recognition system must consider the level of paralysis exhibited by the patient because there is different behavior between healthy and paralyzed facial organs. The recognition systems must be capable of discerning such behavior. The analysis of facial paralysis from images requires new data and models to overcome the bias produced by the use of healthy people photographs in the design, training, and testing of algorithms. Improvements to the methodology would require more data from palsy patients with labeled facial expressions. The code to compute our facial features and the trained models is available upon request.

## Figures and Tables

**Table 1:** General description of the facial gestures.

Facial Gesture	Face Region		
	Eyebrows	Eyes	Mouth
Neutral face	No movement	No movement	No movement
Eyebrow elevation	Inner brow raiser (AU1) and outer brow raiser (AU2)	Irrelevant	Irrelevant
Eye closure	Irrelevant	Eyes closed (AU43)	Not important
Wide-open smile	Irrelevant	Irrelevant	Lip corner puller (AU12), lips part (AU25) and jaw drop (AU26)
Closed mouth smile	Irrelevant	Irrelevant	Lip corner puller (AU12) and lip stretcher (AU20)
Pucker	Irrelevant	Irrelevant	Lip puckerer (AU18)

**Table 2:** Facial features introduced by Parra et al. in [16].

No.	Element	Formula	Description
*f0*	Eyebrows	|∠(P0,P9)|	*Y*-axis position of the left and right sides.
*f1*	Eyebrows	|∠(P2,P7)|	*Y*-axis position of the left and right sides.
*f2*	Eyebrows	|∠(P4,P5)|	*Y*-axis position of the left and right sides.
*f3*	Eyebrows	max(L/M,M/L)	Eyebrows’ height points ratio.
*f4*	Eyebrows	m(P0,P9)	*Y*-axis position of the eyebrows.
*f5*	Eyebrows	m(P2,P7)	*Y*-axis position of the eyebrows.
*f6*	Eyebrows	m(P4,P5)	*Y*-axis position of the eyebrows.
*f7*	Eyes	|∠(P10,P19)|	*Y*-axis position of the eyes’ corner.
*f8*	Eyes	$\max(B_{l}/B_{r},B_{r}/B_{l})$	Eyes’ width ratio.
*f9*	Eyes	max(D/E,E/D)	Eye to head side distance ratio.
*f10*	Eyes	max(H/I,I/H)	Eye to nose distance ratio.
*f11*	Eyes	max(N/O,O/N)	Eyes’ average opening ratio.
*f12*	Eyes	$\max(N_{l}/O_{r},O_{r}/N_{l})$	Eyes’ outer opening ratio.
*f13*	Eyes	$\max(N_{r}/O_{l},O_{l}/N_{r})$	Eyes’ inner opening ratio.
*f14*	Mouth	|∠(P28,P34)|	*Y*-axis position of the corners.
*f15*	Mouth	max(F/G,G/F)	Eye to mouth distance ratio.
*f16*	Mouth	$\max(P_{l}/Q_{l},Q_{l}/P_{l})$	Mouth’s outer opening ratio.
*f17*	Mouth	$\max(P_{u}/Q_{u},Q_{u}/P_{u})$	Mouth’s inner opening ratio.
*f18*	Mouth	$\max(V_{l}/A,V_{r}/A)$	Mouth’s half width and head width ratio.
*f19*	Mouth	$\max(P_{l}/W,Q_{l}/W)$	Mouth’s outer height and its width ratio.
*f20*	Mouth	$\max(P_{u}/W,Q_{u}/W)$	Mouth’s inner height and its width ratio.
*f21*	Mouth	$\max(W_{l}/W,W_{r}/W)$	Mouth’s half perimeter and its width ratio.
*f22*	Nose	|∠(P23,P27)|	*Y*-axis position of the nose.
*f23*	Combined	|∠(P22,P37)|	*Y*-axis position of the nose and mouth.
*f24*	Combined	max(J/K,K/J)	Nose-to-mouth distance ratio.
*f25*	Combined	max(T/A,U/A)	Eyebrows-to-mouth distance and the head’s width ratio.
*f26*	Combined	max(R/A,S/A)	Eyebrows-to-mouth distance and the head’s width ratio.
*f27*	Combined	C/A	Mouth-to-chin distance and the head’s width ratio.
*f28*	Combined	X/A	Nose-to-mouth distance and the head’s width ratio.

In *f3*, *L* and *M* are the average heights of all the points in the left and right eyebrows, respectively. In *f11*, $N=(N_{l}+N_{r})/2$, similarly, $O=(O_{l}+O_{r})/2$. In *f19*, *f20* and *f21*, *W* is the distance depicted in Figure 3d, with the perimeter values $W_{l}$ and $W_{r}$ calculated as $W_{l}=\bar{S}\left(P28,P29,P30,P31,P37,P38,P39\right)$ and $W_{r}=\bar{S}\left(P31,P32,P33,P34,P35,P36,P37\right)$.

**Table 3:** The results per fold of the facial gesture recognition system.

n-Fold	Correct	Errors	Accuracy
1-fold	509	51	90.89%
2-fold	506	54	90.36%
3-fold	501	59	89.46%
4-fold	507	53	90.54%
five-fold	504	56	90.00%
Overall	2527	273	90.25%

**Table 4:** The results per class of the facial gesture recognition system.

Class	Recall	Precision	F1-Score
Class 0	83.71%	78.55%	81.05%
Class 1	86.57%	89.38%	87.95%
Class 2	89.43%	91.92%	90.66%
Class 3	97.12%	92.84%	94.93%
Class 4	83.52%	86.86%	85.15%
Class 5	88.57%	95.68%	91.99%

Class 0 refers to neutral face, class 1 to eyebrow elevation, class 2 to eye closure, class 3 to wide-open smile, class 4 to closed mouth smile, and class 5 to pucker.

**Table 5:** The results per class for each level of paralysis of the facial gesture recognition system.

Test	Class 0	Class 1	Class 2	Class 3	Class 4	Class 5	Overall
Normal	75.71%	87.14%	86.43%	98.76%	91.84%	97.14%	90.71%
Near-normal	87.14%	94.29%	85.71%	98.81%	80.95%	91.43%	91.25%
Mild	71.43%	80.0%	80.0%	98.41%	76.19%	97.14%	87.14%
Moderate	87.14%	95.71%	87.86%	97.80%	89.29%	81.43%	91.25%
Severe	88.57%	85.71%	86.43%	96.89%	93.88%	91.43%	90.89%
Complete	91.43%	95.71%	93.57%	100%	100%	100%	96.79%
Average	83.57%	89.76%	86.67%	98.44%	88.69%	93.09%	91.34%

Class 0 refers to neutral face, class 1 to eyebrow elevation, class 2 to eye closure, class 3 to wide-open smile, class 4 to closed mouth smile, and class 5 to pucker.

## Data Availability

There is no available data.
